# Assessment of Ki-67 proliferation index with deep learning in DCIS (ductal carcinoma in situ)

**DOI:** 10.1038/s41598-022-06555-3

**Published:** 2022-02-24

**Authors:** Lukasz Fulawka, Jakub Blaszczyk, Martin Tabakov, Agnieszka Halon

**Affiliations:** 1 Molecular Pathology Centre Cellgen, ul. Piwna 13, 50-353 Wroclaw, Poland; 2 Department of Computational Intelligence, Wroclaw University of Science and Technology, wybrzeże Wyspiańskiego 27, 50-370 Wrocław, Poland; 3 Department of General and Experimental Pathology, Wroclaw Medical University, ul. Borowska 213, 50-556 Wroclaw, Poland

**Keywords:** Biomarkers, Mathematics and computing, Cancer

## Abstract

The proliferation index (PI) is crucial in histopathologic diagnostics, in particular tumors. It is calculated based on Ki-67 protein expression by immunohistochemistry. PI is routinely evaluated by a visual assessment of the sample by a pathologist. However, this approach is far from ideal due to its poor intra- and interobserver variability and time-consuming. These factors force the community to seek out more precise solutions. Virtual pathology as being increasingly popular in diagnostics, armed with artificial intelligence, may potentially address this issue. The proposed solution calculates the Ki-67 proliferation index by utilizing a deep learning model and fuzzy-set interpretations for hot-spots detection. The obtained region-of-interest is then used to segment relevant cells via classical methods of image processing. The index value is approximated by relating the total surface area occupied by immunopositive cells to the total surface area of relevant cells. The achieved results are compared to the manual calculation of the Ki-67 index made by a domain expert. To increase results reliability, we trained several models in a threefold manner and compared the impact of different hyper-parameters. Our best-proposed method estimates PI with 0.024 mean absolute error, which gives a significant advantage over the current state-of-the-art solution.

## Introduction

The recent state-of-the-art computer vision systems are based mostly on deep learning models, which are part of the well-known machine learning. As deep models, the convolutional neural networks (CNN) are essential in image segmentation, classification, or recognition. Medical image analysis became an important domain of CNN applications. Recently, deep learning techniques mostly based on CNN models were applied successfully in order to provide Ki-67 index evaluation as well. Gamma mixture model with expectation–maximization applied in the deep learning process was proposed in^1^, a model called PathoNet for Ki-67 immunostained cell detection and classification was proposed in^2^, MobileUnet model used for segmentation of Ki-67 images was introduced in^3^ or other dedicated convolutional neural networks models^4,5^. Nevertheless, the success of any CNN model is determined by the correctly prepared training sets or the histology preparation used. In our research, we used whole slide images (WSI) of ductal carcinoma in situ of the breast (DCIS), which are now the standard data source in digital pathology.

### Ki-67 protein and proliferation index (PI)

Accelerated and uncontrolled proliferation is one of the main features defining malignancy. The well-established marker of proliferation in the routine histopathological examination is the Ki-67 antigen, detected by immunohistochemistry^6–8^. It is a nuclear antigen detected in all phases of the cell cycle other than the G0 phase (resting cells)^6^. Proliferation activity is determined as the ratio of Ki-67-positive cells (given as a percentage), commonly known as proliferation index (PI) (Eq. 1).

1 $$PI={\frac{{N}_{Ki-67\left(+\right)}}{{N}_{Ki-67\left(+\right)}{+N}_{Ki-67\left(-\right)}}}x 100\%$$

Proliferation index (PI), where N_Ki-67(+)_—number of Ki-67-positive cells, N_Ki-67(−)_—number of Ki-67-negative cells.

Evaluation of this parameter is conducted in routine histopathological examination of cases in some types of conditions, especially tumors. PI is considered a prognostic or predictive biomarker in certain types of cancer. It plays a significant role in surrogate biological subtyping of invasive breast cancer (IBC). These types determine the line of anticancer therapy (chemotherapy vs. hormonal therapy vs. targeted therapy) in IBC^6,8–10^. There are few studies investigating the role of PI as a prognostic factor in DCIS.

PI is routinely evaluated by visual assessment (VA) of the sample by a pathologist. The most commonly applied method is eyeballing (EB)^9^. However, this approach is far from ideal due to its poor reproducibility. In one of our previous studies, we revealed significant differences between pathologists evaluating PI in breast cancer slides by their routine daily mode i.e. EB (reaching up to 57%)^9^. These factors raise uncertainty about the role of PI in some experts.

### Proliferation index (PI) assessment by digital image analysis (DIA)

The most precise way of PI assessment is one-by-one nuclei counting. Obviously, it is not possible to use only a standard microscope and tissue slides. A grid is used by a few of our colleagues from other hospitals but it has not been mentioned in any paper studied by us. In practice, the only way to count nuclei is by using software, which enables pointing single cells on a digital image, or region of interest (ROI) selected in a virtual slide^10^. It should be noted that further in this article this term is highlighted as pathological ROI (pROI) to distinguish it from the abbreviation ROI in image analysis, used later in this article. However, pointing single cells is laborious and time-consuming. Therefore, it is not useful in laboratories with many cases, especially tumors, being diagnosed but rather for research purposes.

The above factors force the community to seek out more precise solutions. The use of digital image analysis (DIA) may potentially address this issue. Most of the commercial microscopic image analysis platforms have nuclear counting options included. The examples include Aperio (Leica Biosystems), TissuemorphKP (Visiopharm), GenASIs (Applied Spectral Imaging), NuclearQuant (3DHISTECH) and Virtuoso (Ventana, Roche)^10^. Our study aimed to build accurate free-for-use software for PI calculation with CNN model and dedicated image processing. We have compared our results to those obtained by the one-by-one nuclei counting method, which was used as a gold standard (the term synonymous with ground truth) and the current state-of-the-art solution called PathoNet, which introduces its own backend neural network, a pipeline for segmenting tumor cells and compares to other solutions being variations of the recent methods used as a backend in their proposed pipeline^2^.

### Ductal carcinoma in situ (DCIS)

The widespread introduction of mammographic screening has contributed to a significant increase in ductal carcinoma in situ of the breast (DCIS) incidence. This lesion is diagnosed during a histopathological examination of tissue samples usually from biopsies of suspicious microcalcifications detected on a mammography^11–13^. Detection of DCIS is of exceptional value because this lesion is a non-obligate precursor of invasive breast carcinoma (IBC). Diagnosis of DCIS indicates a 10 times higher risk of development of IBC if left untreated^14^.

There are a variety of prognostic factors in DCIS, which belong to clinical and histopathologic features such as patient age, the extent of DCIS, nuclear grade, comedo necrosis, multifocality, margin status, and mode of presentation^14^. Among them, nuclear grade and comedo necrosis are the strongest histopathologic prognostic markers^14^. The prognostic factors are of significant importance because 50% of DCIS recurrence presents as invasive cancer^15^.

In line with the current knowledge, DCIS is a heterogeneous group of cancers with distinct molecular features. Therefore the paradigm that “one approach fits all” in patients with DCIS does not work anymore^15^. There is a significant demand for more objective ways of classification and risk stratification of DCIS. It has been revealed that the same intrinsic molecular subtypes that exist in invasive breast carcinoma can be also identifiable in DCIS^14^.

As mentioned before, the knowledge about the role of PI as a prognostic factor in DCIS is still limited due to the low number of studies. One of them is a meta-analysis by Poulakaki et al. who found an association between high PI and risk of recurrence in women with DCIS (66% increase of invasive or noninvasive recurrence rates)^16^.

## Methods

### Tissue material and processing

The tissue slides derive from archival formalin-fixed paraffin-embedded (FFPE) tissue samples from 95 female patients with ductal carcinoma in situ (DCIS) who have undergone minimally invasive vacuum-assisted percutaneous breast biopsy in Breast Unit, Lower Silesian Oncology Center (Wroclaw). The use of the material has been approved by the Director of Lower Silesian Oncology Center. Additionally archival hematoxylin–eosin slides for each case were retrieved to be assessed as a “second look” by the two board-certified pathologists experienced in breast cancer (AH and LF). The IHC reaction was conducted with rabbit monoclonal ready-to-use (2 µg/mL) antibody against human Ki-67 antigen (Clone 30-9) and ultraView Universal DAB Detection Kit (Ventana, Tucson, AZ, USA). The procedure was performed automatically using Benchmark XT (Ventana, Tucson, AZ, USA), in line with the producer’s manual. The 4-µm-thick paraffin sections were cut and mounted on SuperFrost Slides (Thermo Fisher Scientific, Gerhard Menzel GmbH, Braunschweig, Germany).

Research was conducted in accordance with local guidelines and regulations. The study was approved by local ethics committee (Bioethics Committee Wroclaw Medical University).

### PI calculation algorithm

**Figure 1:**
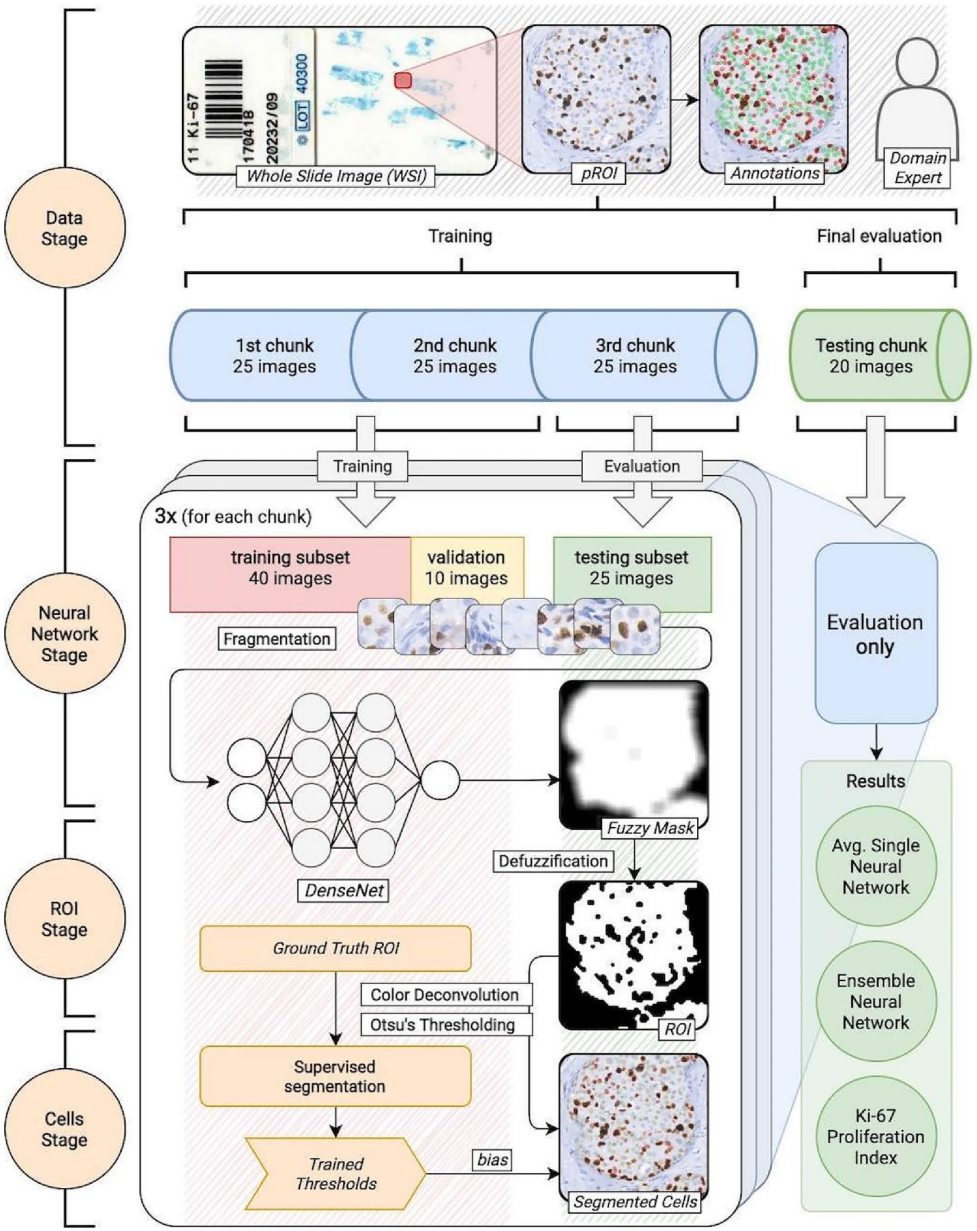
The complete experiment, divided into 4 stages. The primary source of data is a collection of 95 whole slide images (WSI). For each case, one hot-spot was selected and designated as a rectangular area, i.e. pROI (pathological region of interest) by a domain expert. Then, the images are annotated by the same expert and split into 4 chunks. The First 3 chunks are used to train and evaluate models (in a k-fold manner). The last chunk is used only to evaluate. This means, that at the end, we had results from 45 images for a single model (20 images from testing chunk + 25 from the one chunk used as a testing subset) and 20 images for an ensemble model (only from the testing chunk because other images have been utilized by at least one componential model).

The main idea lying under our research is to primarily identify regions of interest (ROI) decisive for Ki-67 index calculation. For this purpose, we apply a convolutional neural network (CNN) model with the fuzzy interpretation of obtained masks and further, image segmentation of tumor cells. The applied CNN model is based on the DenseNet architecture^17^ using Tensorflow^18^ and Keras^19^. The entire experiment is divided into the following stages (Fig. 1):

- **Data stage**: acquiring and annotating histopathology images followed by initial pre-processing,
- **Neural network stage**: k-fold training and evaluating of CNN models,
- **ROI stage**: fuzzy interpretation of the neural network predictions,
- **Cells stage**: tumor cells segmentation with calculating Ki-67 proliferation index.

The complete method and experiment code is publicly available at https://github.com/jamebs/ki67.

### Data

The primary database is a collection of 95 whole slide images (WSI) of ductal carcinoma in situ (DCIS). The WSI were obtained using the two slide scanners: ScanScope CS (Aperio ePathology, Leica Biosystems Imaging, Vista, CA, USA) and Pannoramic 250 (3DHistech Ltd., Budapest, Hungary). The hot-spots, defined here as areas of elevated Ki-67 protein expression, i.e. locally increased proliferation index, were designated by a domain expert—board-certified pathologist (LF). For each case, one hot-spot was selected and designated as a rectangular area, i.e. pROI (pathological region of interest) (Fig. 1/data stage). The selection criteria for pROIs were as follows: representativeness in terms of morphology, the highest image sharpness, possible lack of artifacts, absence of unspecified cytoplasmic staining pattern and extracellular reaction, possible avoidance of tissue edges. It does not mean that selected pROIs do not contain such areas. Our aim was to select areas with as much as possible tumor cells which can be properly marked by domain expert. It gives the algorithm more data to train. Moreover the percentage of neoplastic cells relative to non-neoplastic cells and extracellular matrix was not taken into account to train and validate the algorithm and test its performance in real clinical conditions. The size of pROIs varies from 736 × 1216 pixels (0.895 Mpix) to 9488 × 4832 pixels (45.846 Mpix) with an average size of 9.705 Mpix.

**Figure 2:**
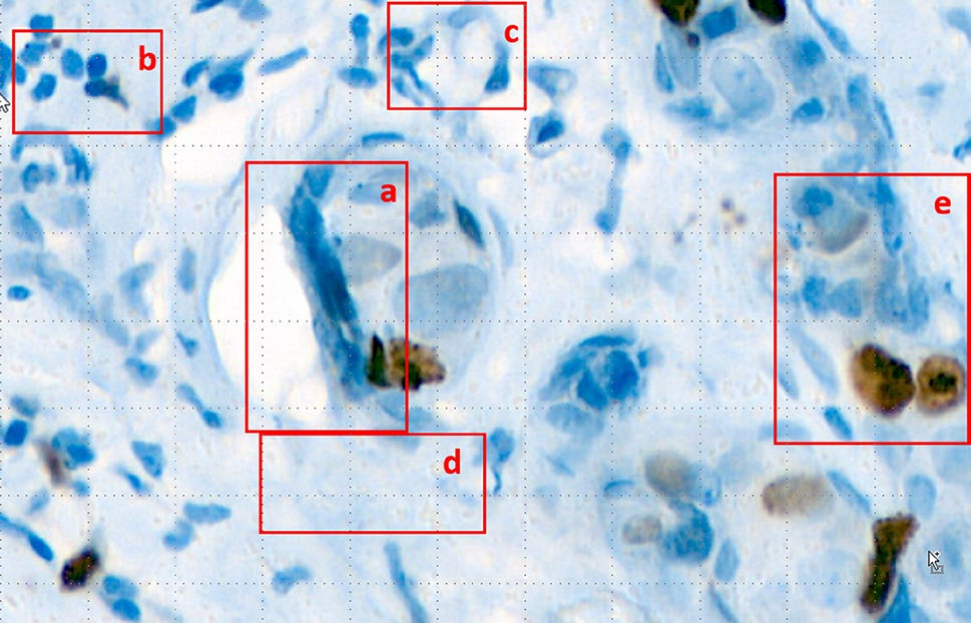
Example of IHC slide. In the red regions there are marked: (**a**) artifacts, (**b**) lymphocytes, (**c**) blood vessels, (**d**) collagen fibers and (**e**) tumour cells with high and low expression of Ki-67 protein (our assumed ROI).

The images of pROIs were saved as tiff/png files with lossless compression. They are stored as 3-channel (red–green–blue) digital images. Each Ki-67 slide is composed of 2 stains: diaminobenzidine (DAB) for positive nuclei and hematoxylin for all nuclei and background. In traditional RGB color palettes, they are depicted as shades of browns and blues respectively. However, staining is not limited to cancer cells. It has to be remembered that cancer tissue consists of cancer cells as well as stromal components, mainly fibroblasts, collagen fibers, immune cells (overwhelmingly lymphocytes), and blood vessels (Fig. 2). Moreover, tissue processing produces artifacts that make image analysis much more difficult.

The annotation process was done by a domain expert (LF). For this purpose, we used ImageJ with the Cell Counter plug-in^20–22^. The methodology was described previously by us^10,23^. The diaminobenzidine (DAB) and hematoxylin-stained neoplastic nuclei were annotated in the pROIs (Fig. 1). The Ki-67-positive nuclei were taken into account despite the staining intensity, in line with the recommendations from the International Ki-67 in Breast Cancer Working Group^6^. The artifactually changed nuclei were not marked. A total number of 150,592 nuclei were selected, including 26,093 DAB-stained nuclei. On average 1585 (median 1220) nuclei per image were annotated. The annotations were revised and accepted by the second board-certified pathologist (AH). From the data scientist’s point of view, the crucial insight from the expert's annotation procedure is the initial extraction of tumor cell clusters. It means that relevant are only these cells, which are placed together into groups. The minimal quantity of the cells in such groups is very subjective, but single cells in the intercellular space are always skipped.

The ground truth (gold standard) is labeling index, i.e. proliferation index (PI), calculated by dividing the number of DAB-stained neoplastic nuclei by the total number of neoplastic nuclei (the sum of DAB- and hematoxylin-stained nuclei) (Eq. 1). The number of each category was derived from annotations with ImageJ described above.

The complete data set containing 95 annotated images was randomly divided into 4 chunks: 3 training sets of 25 images each and one testing set containing 20 images. These training sets are used in a k-fold training manner of 3 CNN models in the next stage. The last subset is used only for testing purposes, so none of these 20 images have been ever used in any intermediate stage.

Summarizing—the results of this stage are annotated and partitioned all pROIs of the IHC image data set.

### Neural network classification

This phase is focused on utilizing deep learning methods. The implemented CNN classification procedure aims to identify automatically the whole set of image ROI, necessary for further calculation of the ki-67 index. Within this procedure, the deep neural network highlights regions with a high density of relevant cells and discards any stain artifacts, which can mislead the segmentation phase.

Each CNN model (based on the same architecture) is trained on 2 chunks (50 images), which are randomly divided into training and validating subsets in ratio 4:1. The third chunk with 25 images—different for respective models—is only used to obtain results after completing the whole training. It is important to know that the IHC images are processed with a sliding window to generate a sufficient number of examples. Therefore, the inputs of the CNN are assumed as image fragments, which partially overlap. There are 2 motivations supporting this approach. Firstly, we would like to handle cells that might be only partially presented in a considered fragment. Secondly, we would like to harden the ROI against the noise, which might lead to fragment misclassification. Using the proper size of the window is crucial for the whole task, however, its right value is unknown. Therefore, we performed the independent experiments for 3 different quadratic windows: 48, 96, and 192 pixels length. The window stride is set to 16 pixels for each model evaluation, which is a compromise between classification precision and fragment diversity. For the training process, we increase stride to the window size to reduce the risk of overfitting. Each model is trained with thousands of fragments: from around 8000 for 192-pixels windows up to 150,000 for 48-pixels windows. The CNN performs a binary classification task that assigns positive labels to fragments with at least one relevant cell and negative labels otherwise.

The models are based on the DenseNet121^17^ architecture with a reduced batch normalization momentum to 0.1. The training is performed with Adam optimizer (with learning rate set to 0.001) and it is based on the binary cross-entropy loss function. Each model is trained with a batch size of 128 samples for 50 epochs. To overcome the overfitting problem, we apply the mechanism evaluating the model after each epoch. The model is saved only when it has improved (the loss-value has decreased).

After training all 3 models of the same window size, we combine them to create an ensemble classifier similar to the bagging method^24^. It works like a voting system, which gets results solely from each model and assigns the fragments label established by at least 2 component models (independently per fragment). Each composing model is learned on a different fragments subset, which makes it sensitive to various features (and noise). Combining them, we reduce the model's uncertainty. The ensemble classifier is evaluated only on 20 slides from the testing chunk because all 3 training chunks have been used to create that classifier.

Summarizing—the result of this stage is 3 base models and 1 ensemble classificator with predictions made for adequate testing images.

### ROI defuzzification

**Figure 3:**
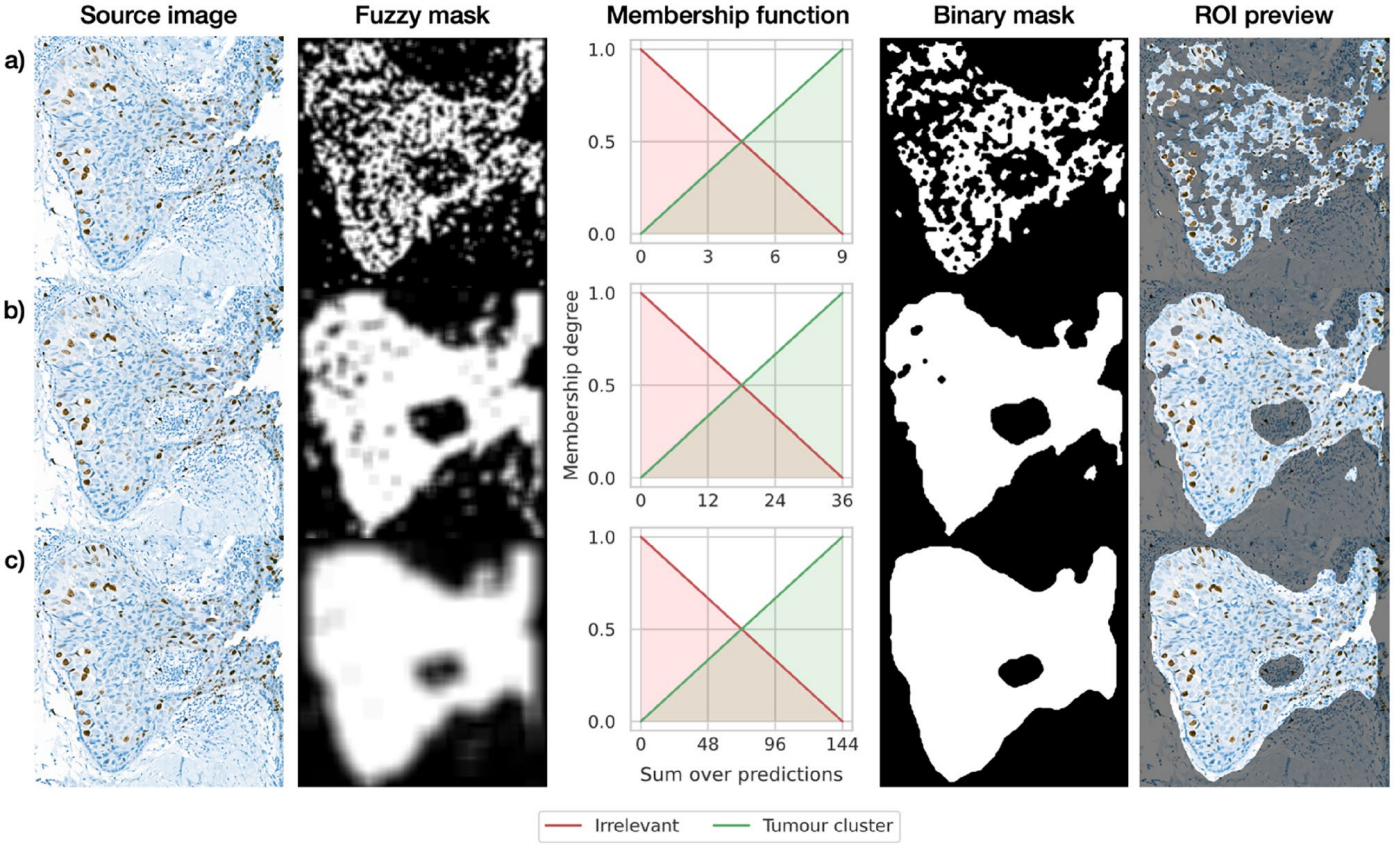
The complete defuzzification process for (**a**) 48-, (**b**) 96-, (**c**) 192-pixels window size applied in the neural network stage.

This phase is focused on producing the binary mask highlighting tumor cell clusters. It should be noted that because of the sliding window applied, the CNN predictions for image fragments overlap. Therefore, we need an additional procedure to provide the final classification decision for each pixel. We introduce the fuzzy interpretation (with respect to fuzzy set theory^25^) of the obtained predictions for this purpose. Namely, every part of the IHC image can be described as a tumor cluster or as an irrelevant part. These two classes have become fuzzy sets with linear membership functions. The image part affiliation (membership degree) is calculated as a sum over all predictions for that part, where the positive label is treated as 1 and the negative as 0. For fragments with size 96 × 96 pixels and stride equals 16 pixels, the result of 0 entirely means irrelevant part, while value 36 (the maximum, which any part can achieve, because 96 px window divided by 16 px stride gives 6 subparts horizontally and 6 subparts vertically)—certain tumor cluster. Finally, all pixels are assigned to the most suitable class in order to get a binary mask highlighting ROI. It should be noted that the above interpretation also hardens against noise misclassifications of the neural networks. Currently, we focus on linear membership function, however, it is not the only solution for that approach. Future work can experiment with more complex functions to leverage ROI segmentation. The complete process on an example image is presented in Fig. 3.

Summarizing—the result of this stage is ROI binary masks for all IHC images (based on an appropriate CNN model).

### Cells segmentation with Ki-67 proliferation index calculation

The identification of relevant cells is critical to provide the Ki-67 index. In this phase, we use a similar approach (approximating PI by cells area) to the one introduced in ImmunoRatio^26^. However, we reduce the segmentation area to the binary ROI from the preceding stage.

Firstly, the slide is converted from the RGB color model into HED via color deconvolution^27^ according to skimage^28^ v0.15 implementation. Then, the highlighted regions are separately processed for brown cells (diaminobenzidine channel) and all relevant cells (hematoxylin channel). At this point, the cells and background are separable within a single channel. It can be approximated to a bimodal distribution of intensity of respective staining. Hence, we can apply Otsu’s thresholding method to cut off cells from the background^29^. This produces masks respectively of brown and all relevant cells. It should be noted that image histogram (and further Otsu’s thresholding) is calculated only for the ROI to omit any staining artifacts, which could bias the intensity distribution.

Most of the current solutions focus on dividing joined cells. The CNN filters out most of the artifacts, however even a single flaw can strongly bias the final score. Therefore, we approximate the proliferation index by comparing the total surface area occupied by brown cells to the total surface area of relevant cells. A similar approach was proposed^30^. With the usage of binary closing and dilatation, this solution reduces artifacts’ impact on the final score. However, this process is very vulnerable to any color deviations, which can mislead the thresholding. To reduce such a risk, we also add threshold biasing. For each training slide, we calculate the average thresholds based on Otsu’s algorithm and ground truth ROI (generated from expert annotations). Then, each chunk is biased with average thresholds calculated from the slides of the complementary chunks. The mechanism is analogous to the CNN training, e.g. the first chunk is biased with thresholds from two other training chunks.

Summarizing—the result of this stage is the final Ki-67 proliferation index.

### PathoNet estimations

To compare our solution to the current state-of-the-art method, we take into account a PathoNet architecture with a watershed algorithm^2^, which is the most recent and the most promising method presented in 2021. By completely retraining the model, we minimize the risk of corrupting results by the incoherence to data used in the train and validation phases. Thus, the model is tuned from the scratch only on our images: 75 from all training chunks and evaluated on 20 testing slides.

We took the crucial parts of the logic from the PathoNet authors’ implementation published on GitHub (https://github.com/SHIDCenter/PathoNet). The model has been trained with the original hyperparameters: 30 epochs with 16 samples in a batch and the initial learning rate equals 0.01. The samples with the size 256 × 256 pixels (the same as in the original article) were drawn from 75 images from all training chunks and split into 80% for train and 20% for validation. We noticed that the PathoNet algorithm is very sensitive for input data. Therefore, we have also adapted it to our data by training separate models for each of two classes, increasing “gaussian size” (used for pre-processing ground truth labels), lowering threshold, and reducing the number of negative samples (fragments without labels). The rest of the hyperparameters remain unchanged.

We trained and evaluated following the original approach (without our modifications) and then with our adaptations. The second approach achieved better results for our data, therefore we compared our proposed method to them.

### Ethics approval

Research was conducted in accordance with local guidelines and regulations. The study was approved by local ethics committee (Bioethics Committee Wroclaw Medical University).

## Results

We performed several experiments with various configurations of the presented method. The experiments can be divided into two groups based on compared aspects: measuring neural network effectiveness and proliferation index exactness.

### Neural networks effectiveness

The experiments related to the neural networks focus on evaluating how accurately a model classifies fragments of IHC images. For this purpose, we measure each model's results with 4 metrics: accuracy, precision, recall, and F1-score (Eq. 2).

2 $$\begin{gathered} Accuracy = \frac{{TP + TN}}{{TP + FP + TN + FN}}\quad Precision = \frac{{TP}}{{TP + FP}} \hfill \\ Recall = \frac{{TP}}{{TP + FN}}\quad F_{1} score = \frac{{2*Precision*Recall}}{{Precision + Recall}} \hfill \\ \end{gathered}$$

Models’ evaluation metrics, where TP—True positive, TN—true negative, FP—false positive (type I error), FN—false negative (type II error).

**Figure 4:**
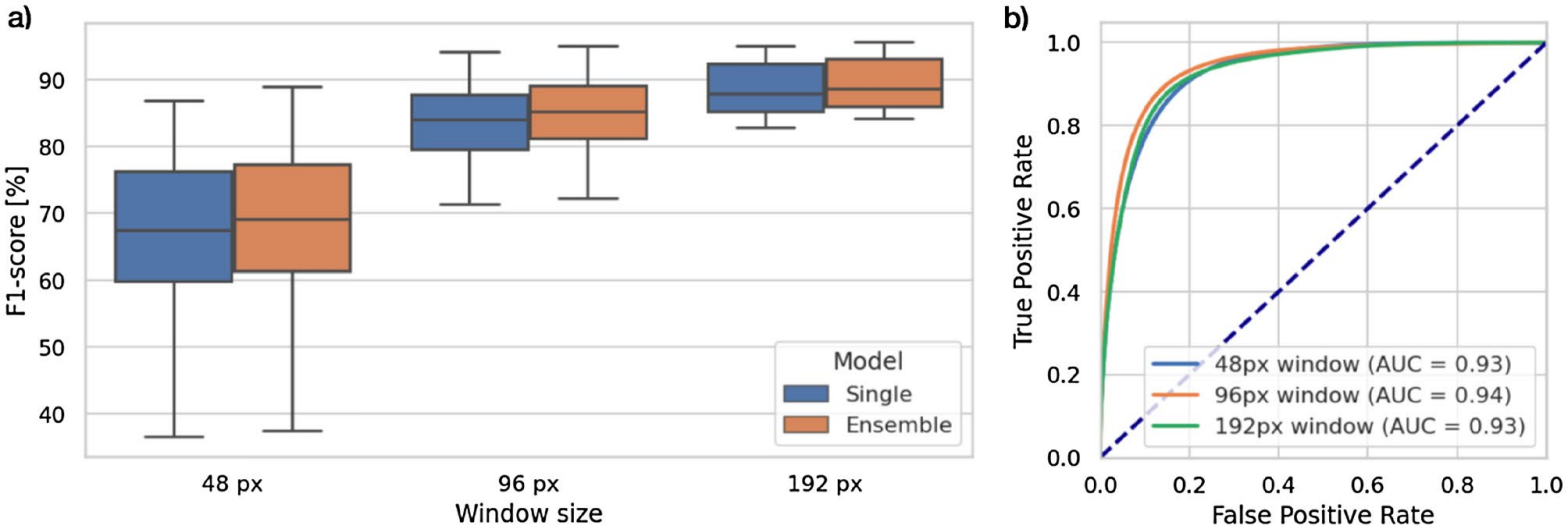
The results of fragments classification: (**a**) F1-score medians and their distributions of compared methods evaluated on 20 images from the testing chunk, (**b**) AUROC (area under the receiver operating characteristics) of the component models (“single”) grouped by the window size.

The experiment is designed to verify two assumptions: (1) the ROI is detected more accurately when applying the bigger window and (2) the ensemble classifier is more accurate than the componential model. For this purpose, we chose 3 window sizes: 48, 96, and 192 pixels (the smaller ones will barely cover a single cell while the bigger ones will contain dozens of them). For the second assumption, we decided to train (in a k-fold manner) and combine 3 models. The results are presented in Table 1 and Fig. 4.

**Table 1:** The collective comparison of fragments classification of the examined models.

Subset	Model	Window size (px)	Accuracy (%)	Recall (%)	Precision (%)	F1-score (%)
75 images (1–3 chunks)	Single (average of 3 models)	48	88.847	74.195	78.510	74.921
		96	89.022	83.985	88.004	84.401
		192	89.234	86.803	92.953	88.678
20 images (4th chunk)	Single (average of 3 models)	48	84.832	66.865	72.323	65.938
		96	83.360	78.528	82.698	76.271
		192	81.942	79.955	87.039	79.742
	Ensemble	48	84.668	66.250	73.792	65.814
		96	83.758	78.498	83.746	76.214
		192	82.584	80.548	89.605	80.323

The presented values are averaged within the indicated subset. Each subset’s image was evaluated by models, which had not been trained on it (as described in the “Data” section).

### Ki-67 proliferation index exactness

The experiments related to the Ki-67 proliferation index focus on comparing the human estimations with the corresponding ones obtained automatically. They put together 4 methods:

- Basic—skips NN stage and performs segmentation on the whole image (referential method, to which we compared our solution)
- PathoNet—calculates the proliferation index completely with a method presented by Negahbani et al.^2^.
- ROI (our)—uses NN-based ROI detection and performs segmentation only within that area (for both a single model and ensemble one)
- ROI + Bias (our)—the above method improved by adding thresholds bias at the segmentation stage

**Figure 5:**
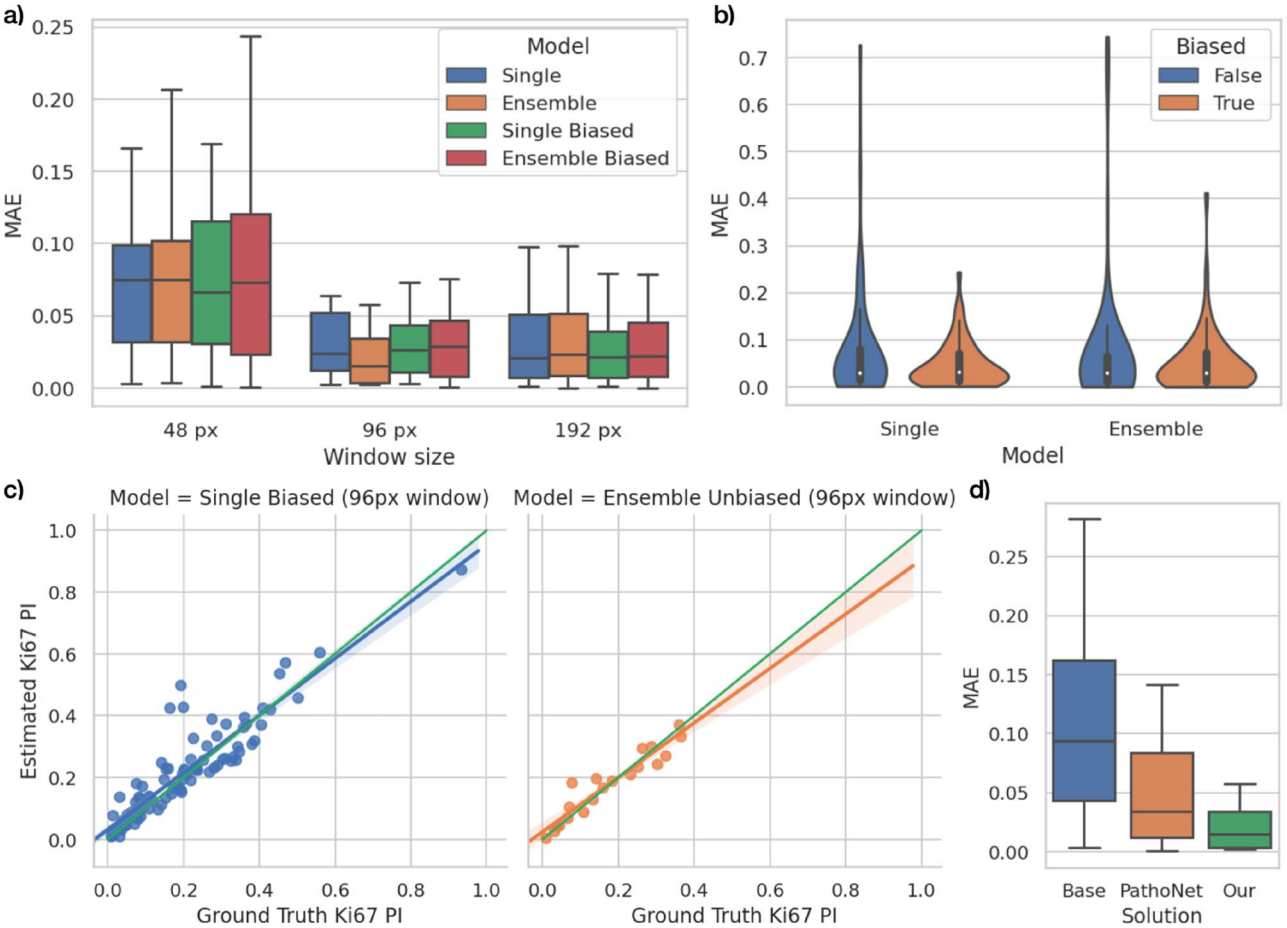
The results of Ki-67 proliferation index estimations: (**a**) presents MAE medians and their distribution for a subset of 20 images with excluded outliers, (**b**) shows the impact of the applied biasing onto reducing outstanding Ki67 PI errors, (**c**) sets linear regression from the best methods (“single biased” and “ensemble unbiased”), (**d**) comparing our best solution (ensemble 96 px-window unbiased) with PathoNet and the “base” approach.

All methods are examined against human-based annotations (manually calculated ki-67 proliferation index by the domain expert). The primary metrics used here are Mean Absolute Error (MAE) and Root-Mean-Square Error (RMSE) donated by the Eq. (3). The results are presented in Table 2 and Fig. 5.

3 $$MAE = \frac{1}{N}{\sum }_{i=1}^{N}\left|{y}_{i}-{x}_{i}\right| RMSE = \sqrt{\frac{1}{N}{\sum }_{i=1}^{N}{\left({y}_{i}-{x}_{i}\right)}^{2}}$$

Mean absolute error ($MAE$) and root-mean-square error (RMSE), where $N$ is the number of samples (slides), ${x}_{i}$ is expert’s Ki-67 proliferation index (true value),${y}_{i}$ is a corresponding proliferation index calculated by a given method (predicted value).

**Table 2:** The collective comparison of Ki-67 proliferation index estimations of the examined models.

Subset	Model	Window size (px)	MAE Ki67 PI (95% confidence interval)		RMSE Ki67 PI		No. invalid estimations (MAE Ki67 PI > 0.2)	
			Unbiased	Biased	Unbiased	Biased	Unbiased	Biased
95 images (all chunks)	Base	–	0.168 (0.122–0.214)	–	0.280	–	21	–
	Single (average of 3 models)	48	0.089 (0.061–0.116)	0.072 (0.052–0.091)	0.161	0.119	7	4
		96	0.064 (0.036–0.092)	0.042 (0.032–0.053)	0.152	0.065	7	3
		192	0.070 (0.038–0.102)	0.039 (0.031–0.047)	0.170	0.054	6	1
20 images (4th chunk)	Base	–	0.167 (0.068–0.265)	–	0.264	–	4	–
	PathoNet	–	0.052 (0.030–0.074)	–	0.069	–	0	–
	Single (average of 3 models)	48	0.093 (0.046–0.140)	0.081 (0.050–0.111)	0.135	0.102	2	1
		96	0.058 (0.019–0.097)	0.034 (0.020–0.047)	0.096	0.040	3	0
		192	0.072 (0.000–0.148)	0.028 (0.016–0.040)	0.173	0.037	2	0
	Ensemble	48	0.116 (0.041–0.192)	0.092 (0.046–0.139)	0.195	0.134	3	2
		96	0.024 (0.012–0.037)	0.032 (0.018–0.047)	0.036	0.044	0	0
		192	0.093 (0.000–0.189)	0.028 (0.017–0.040)	0.221	0.038	2	0

The presented values are averaged within the indicated subset. Due to non-negative values of MEA, the lower boundaries of confidence intervals are limited to 0. Each subset’s image was evaluated by models, which had not been trained on it (as described in the “Data” section).

## Discussion

Taking into account the presented experiments and results, we can make a few conclusions.

First of all, applying cell segmentation (and further their area summation) over a neural-network-based ROI significantly improves the final Ki-67 proliferation index estimation (from MAE of around 0.17 to 0.024–0.11 depending on the method). This proves, that initial region discrimination efficiently removes most of the artifacts and irrelevant cells, which increases mismatched estimation—even with a non-perfect fragments classificator. This leads to a second conclusion, that binary classification for automatic detection of tumor cells is feasible, however not as easy as it is supposed to be. The best neural networks achieve an average of 90% accuracy on 75 testing slides. The most problematic aspect becomes cells at edges, which too narrowing area and context within a single fragment lead to an invalid classification. Consequently, the wider context and thus bigger window size improves the model decisiveness (from 75% F1-score for 48 px window up to 89% for 192 px windows) and confirms our assumption. However, it should be noted that larger windows generate less precise ROI and the final tile size should be a trade-off between classification accuracy and ROI precision. For a few of our 95 images, the CNNs are unable to detect any tumor cells. This is caused by a different scale of cells (they are significantly smaller than on other images), which means that the CNN is very sensitive to cell size. For that case, we assumed the negative variant and performed segmentation on the whole image, to keep the comparison on the same subset of images. The results from the second part of the neural networks experiment also prove that the ensemble model achieves higher accuracy than independently evaluated componential models (also referred to as “single”). However, the difference is only slightly visible due to relatively not-complex problems (binary classification) with a minimal number of voters (only 3 componential models). The future work with more voters and a better-suited heuristic for defining the final decision (than accepting the majority) could increase the ensemble model’s role.

But ROI detection and neural network effectiveness is just an intermediate step and the most important are the results and conclusions related to the proliferation index exactness. First of all, they confirm that window size (and ROI’s trade-off) also has an enormous impact on PI estimation correctness (from 0.11 MAE down to 0.02 for ensemble models). This means that the most accurate is splitting an IHC image into 96-pixels rectangular parts with at most a dozen potential cells. Secondly, they prove how important are changes to even a few classification decisions: ensemble models significantly impact MAE. It increases for extremal window sizes but also decreases by around 3 percentage points for preferred window size.

Despite the mean/median of estimated indexes, no less important is the method reliability measured with an invalid estimation quantity (images for which the solution predicts with more than 0.2 absolute error). This criterion reveals the purpose of the methods utilizing Otsu’s threshold biasing. These methods are characterized by a lower number of mismatched cells-background segmentations in most cases and thereby much lower mean proliferation index error (for the single model with 96 px window it lowers MAE from 0.058 to 0.034 and reduces the number of invalid images from 3 to 0). Yet, this method has the drawback of getting slightly worse estimations for initially well-segmented images, which increase median MAE. Taking these all into account, the threshold biasing can be applied when we care more about the solution stability and skipped when we value the estimation accuracy more.

In general, our solution presents as future-proof with the estimation mismatch of 0.024–0.042 (RMSE 0.036–0.065) for models with a 96 px window evaluated on both testing 20 images and the whole dataset (95 images in a k-fold manner). Treating neural network output as a fuzzy representation of ROI detector significantly reduces the impact of non-tumor cells and artifacts into a final cell segmentation. The additional mechanism like threshold biasing can also tune the method to make it more reliable. The most problematic were few images saved on a different scale, which prevents predicting a valid ROI and tumor cells near blobs, which misleads the segmentation. However, future work with the pre-processing operations, changing the network architecture, or improving the defuzzification process could minimize this problem.

The unquestionable advantage of our research compared to PathoNet (state-of-the-art solution) is the high-quality image database. The primary source is a collection of whole slide images of 95 DCIS cases. Each image comes from a different case. Thus our algorithm was trained on a more cross-sectional and diverse material, which reflects routine histopathological diagnostics. The details of material used in PathoNet training were not satisfactorily described. The images acquired with a standard microscope with a CCD camera are currently not a first-choice option. More and more large pathology departments are equipped with slide scanners. Moreover, IHC in our study was performed automatically using high-quality antibodies, reagents, and equipment corresponding to modern standards. The above-mentioned factors reassured the standardization of material used to train our algorithm. Moreover, our method outbreaks the PathoNet by lowering estimation errors from 0.052 to 0.024 (RMSE 0.069–0.036). This shows how important it is to restrict the nuclei segmentation area before calculating the PI (PathoNet lacks this feature).

**Figure 6:**
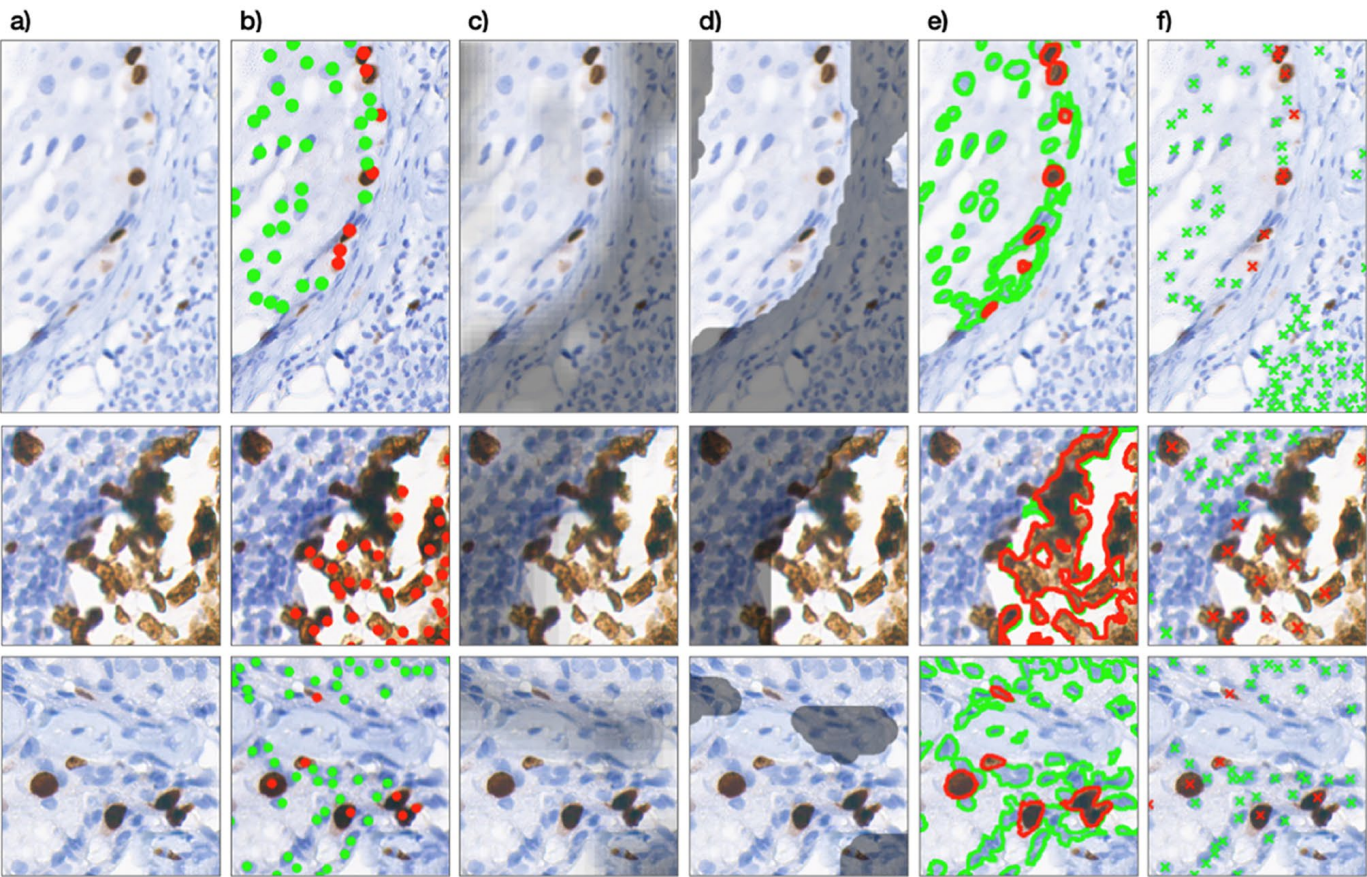
The method outputs overlaying the 3 source images: (**a**) is an input image (**b**) with domain expert’s annotations, (**c**) is a fuzzy output from the neural network stage, (**d**) is a binary mask being a result of the defuzzification process, (**e**) presents selected cells—immunopositive with a red outline and immunonegative with a green one, (**f**) PathoNet estimations as a reference. Our approach selects the nuclei area, while PathoNet the nuclei centers. It should be noted that our solution more accurately rejects lymphocytes and artifacts as non-tumor cells, and therefore, it approximates the proliferation index better. However, for our solution the most problematic are cells near the edges and staining blobs, which might bias the segmentation.

The final advantage of this solution—in contrast to the complete deep-learning-based methods—is high interpretability. Results from each stage can directly overlay the source IHC image and present the ROI, cell segmentation independently for Ki-67-positive and negative cells, and finally the estimated proliferation index, which is presented in Fig. 6. This gives the domain experts an opportunity for quick manual verification and stating the final decision on its own.

This study aimed to develop an algorithm and compare it with the gold standard. In the following phase of our research, we are planning to evaluate the correlation between PI and clinicopathologic parameters in the same group of patients. Although we have gathered a large database of archival data, we believe that the study will be more valuable with prospectively collected survival data. We are currently tracking the medical reports of the study group.

This study aimed to develop an algorithm and compare it with the gold standard. In the following phase of our research, we are planning to evaluate the correlation between PI and clinicopathologic parameters in the same group of patients. Although we have gathered a large database of archival data, we believe that the study will be more valuable with prospectively collected survival data. We are currently tracking the medical reports of the study group.

Digital pathology, as well as molecular pathology, are currently the fastest-growing subdisciplines in pathology. We can see tremendous progress in these fields. We believe that our algorithm will contribute qualitatively to this discipline.

## Data Availability

The complete method and experiment code is publicly available at https://github.com/jamebs/ki67. The tagged images are accessible upon request.
